# Supplementary material for: Cholesterol Activates Cyclic AMP Signaling in Metaplastic Acinar Cells
Source: Metabolites. 2021 Feb 26;11(3):141. doi: 10.3390/metabo11030141 (PMC7996857; doi:10.3390/metabo11030141)
Supplement: Supplementary file 1 [file metabolites-11-00141-s001.pdf]

Figure 1

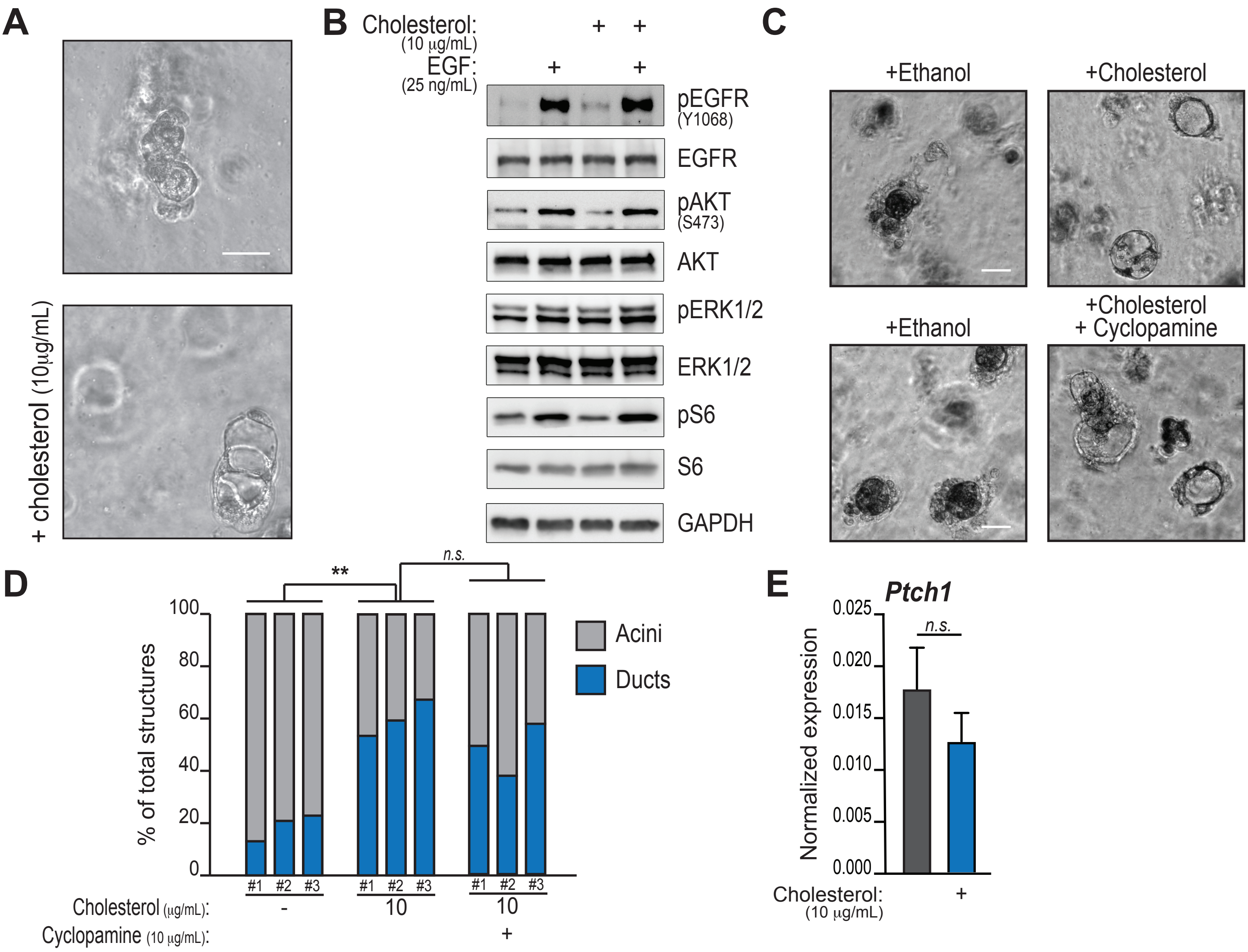

Figure 1. Cholesterol supplementation induces acinar-to-ductal metaplasia (ADM) but does not significantly impact EGFR or Sonic Hedgehog (Shh) signaling. Primary wild-type pancreatic acinar cells were either embedded in a synthetic hydrogel cast (A, C, D, E) or cultured suspended in RPMI medium (B) and stimulated with recombinant EGF (100 ng/mL, unless otherwise indicated). **A,** Cells were treated with free cholesterol (10 µg/mL) or vehicle (ethanol). After 5 days, morphology of cellular clusters was examined. Figure shows representative images of triplicate experiments. Scale bar, 50 µm. **B,** Cells were lysated 20 minutes after EGF addition. Western blotting shows phosphorylation of EGFR and downstream effectors. Representative image of duplicate experiment. **C-D,** Acinar cells were independently isolated from 3 mice (#1, #2, #3) and treated with cholesterol and SHH inhibitor Cyclopamine or vehicle. Morphology was examined after 5 days (**C**) and number of acini/duct structures was quantified by a blinded investigator (in **D**). ******, p<0,01. Scale bar, 50 µm. **E,** qPCR analysis of the *Ptch1* gene in cells treated like in A.

Figure 2

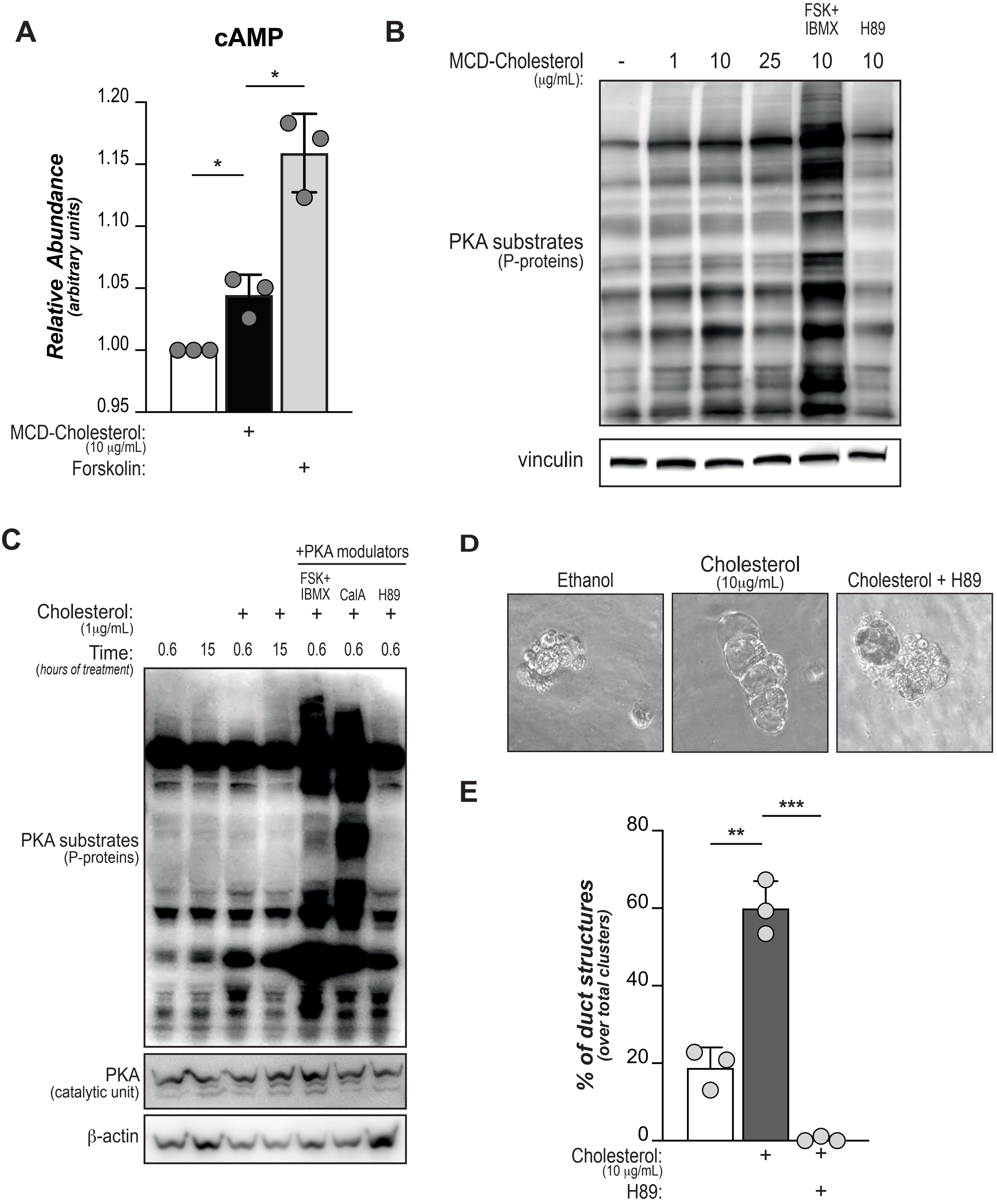

Figure 2. Cholesterol supplementation promotes PKA activation in pancreatic acinar cells. Pancreatic acinar cells were isolated from wild-type animals and supplemented with free- or MCD-bound-cholesterol at indicated concentrations. **A,** cAMP levels were measured in independent acinar explants (n=3). Forskolin was used as positive control. *, p<0,05. **B-C,** Western blotting shows PKA-specific phosphorylation sites in acinar cells treated (B) with increasing concentrations of cholesterol or indicated compounds for 40 minutes or (C) with minimal cholesterol for extended times (indicated). In **C**, total PKA abundance is also shown. **D-E,** Pancreatic acinar cells were isolated from wild-type mice (n=3), embedded in a synthetic hydrogel cast and stimulated with recombinant EGF (100 ng/mL). Cells were treated with free cholesterol (10 mg/mL) ± H89 or vehicle. After 5 days, morphology of cellular clusters was examined. Representative images are shown in D, blinded quantification of duct-like structures in E. **, p<0,01; ***, p<0,001. For all panels, FSK: forskolin (20 μM); CalA: caliculin-A (5 μM); H89: N-[2-p-bromocinnamylamino-ethyl]-5-isoquinolinesulfonamide (30 μM).

Figure 3

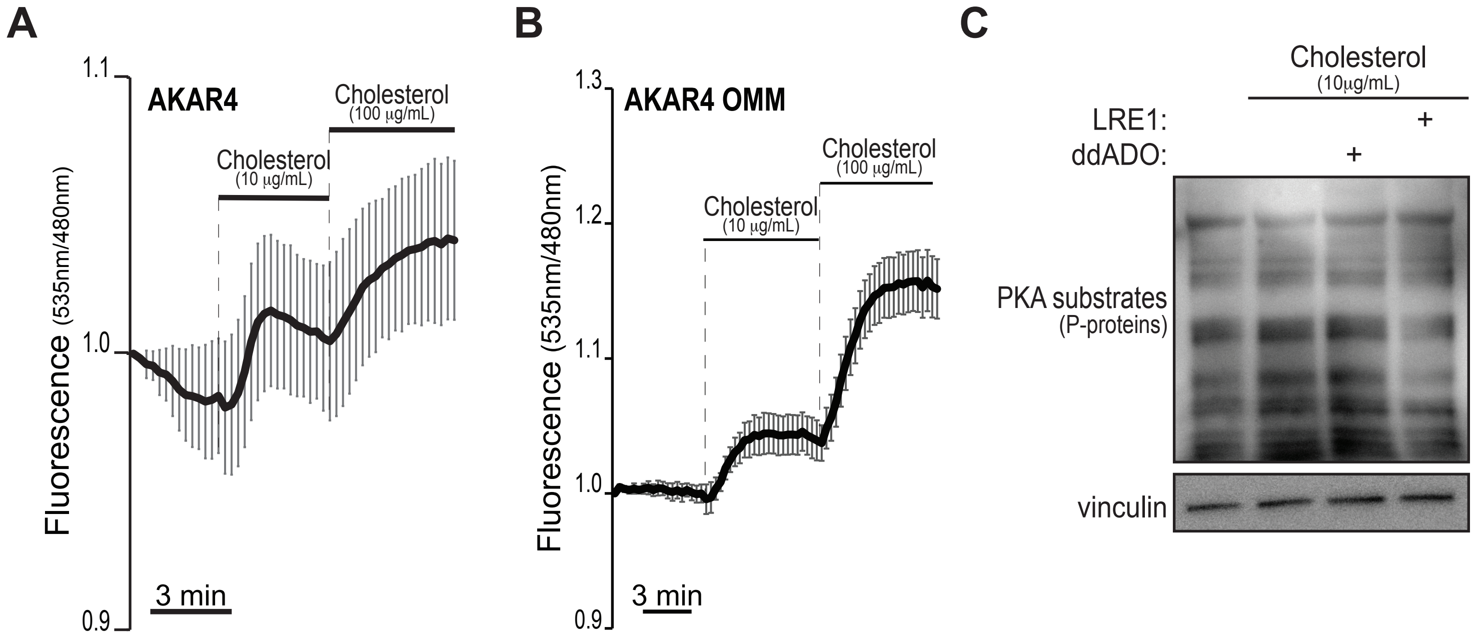

Figure 3. Cholesterol supplementation induces rapid PKA activity predominantly in proximity of sub-cellular organelles. **A-B**, Pancreatic acinar cells were isolated from wild-type animals, infected with adenoviral vectors encoding for the PKA sensors AKAR4 (that senses cytoplasmic PKA activity, panel **A**) and AKAR4-OMM (that targets PKA activity localized on the surface of intracellular organelles, panel **B**). After 36 hours, cells were embedded in a synthetic matrix and imaged. Fluorescence intesities were recorded for several minutes, before and after the addition of indicated amounts of cholesterol. The graphs show FRET ratios over time. Scale bars, 3 minutes. In **C**, primary acinar cells were added cholesterol or vehicle and immediately treated with indicated inhibitors (30 µm, both) for 15 hours. Whole-cell lysates were blotted to detect phosphorylated PKA substrates.

Figure 4

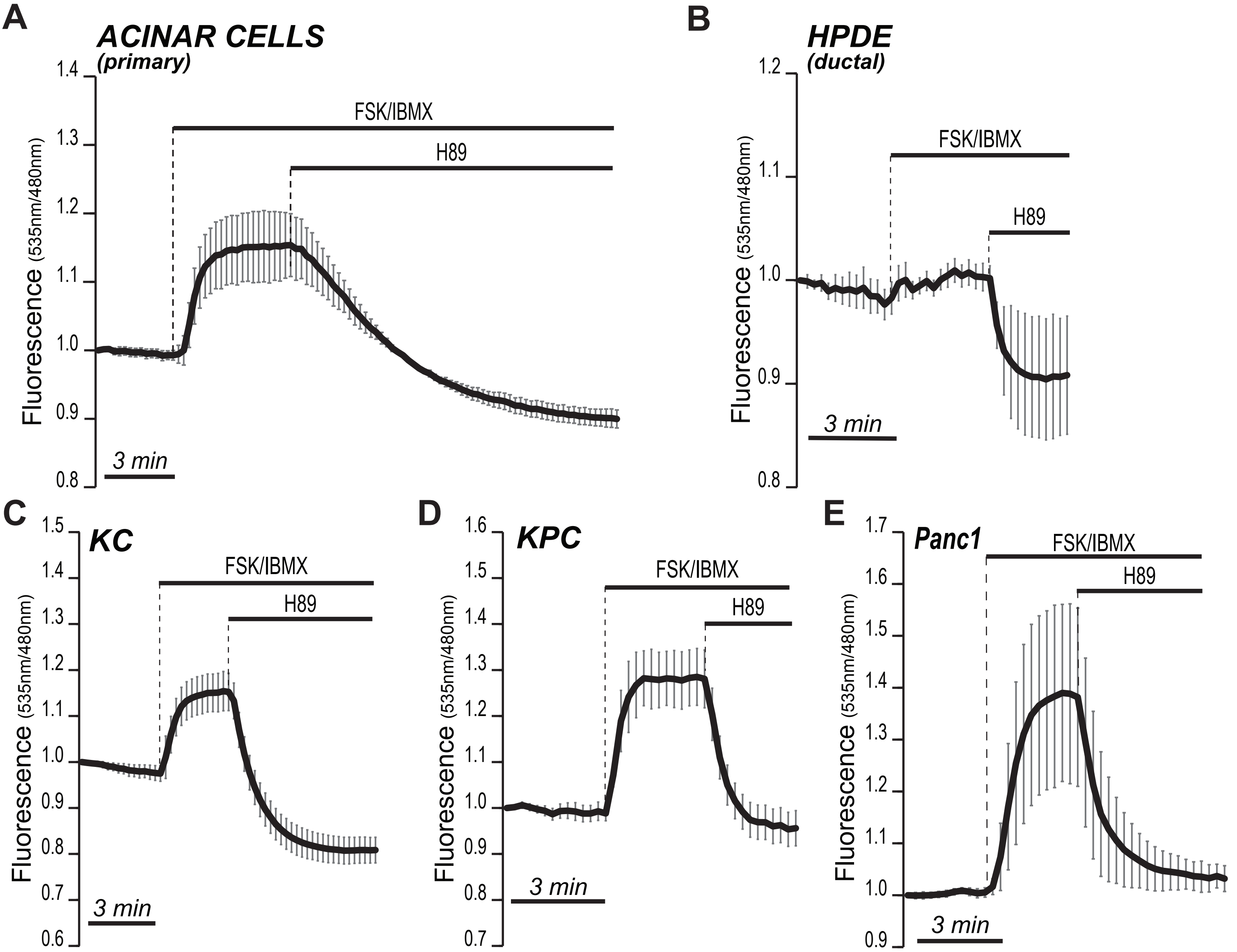

Figure 4. Stage-specific PKA activity in pancreatic cancer cells. Pancreatic epithelial cells (non transformed, ductal and acinar, and tumoral, indicated at the top of each panel) were infected with adenoviral vectors encoding for the PKA sensor AKAR4 and imaged after 36 hours. Fluorescence intesities were recorded for several minutes, before and after the addition of indicated compounds. The graphs show FRET ratios over time. Scale bars, 3 minutes. In **A**, mouse-derived primary acinar cells; in **B**, normal duct cells (commercially-available; HPDE); in **C**, primary mouse cells derived from bona fide pre-malignant lesions (PanIN); in **D-E**, PDA tumor cells, both primary (mouse-derived) and from an established human cell line (Panc1). Graphs are representative of three independent experiments.

Figure S1

A

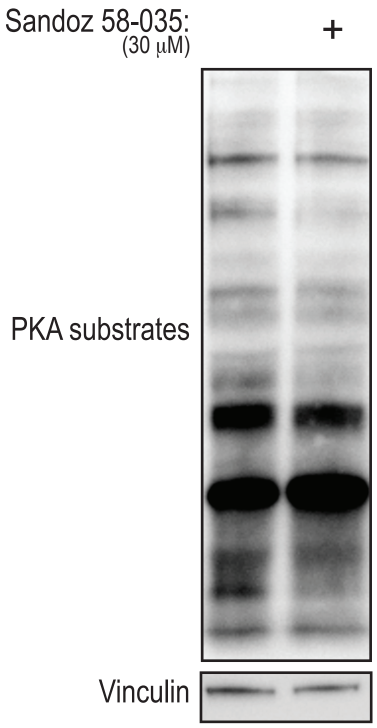

Figure S1. Blockade of cholesterol esterification does not alter PKA activation. Primary acinar explants were treated with Sandoz 58-035 or vehicle (DMSO) for 24 hours. Whole-cell lysates were blotted against phosphorylated PKA substrates.

Figure S2

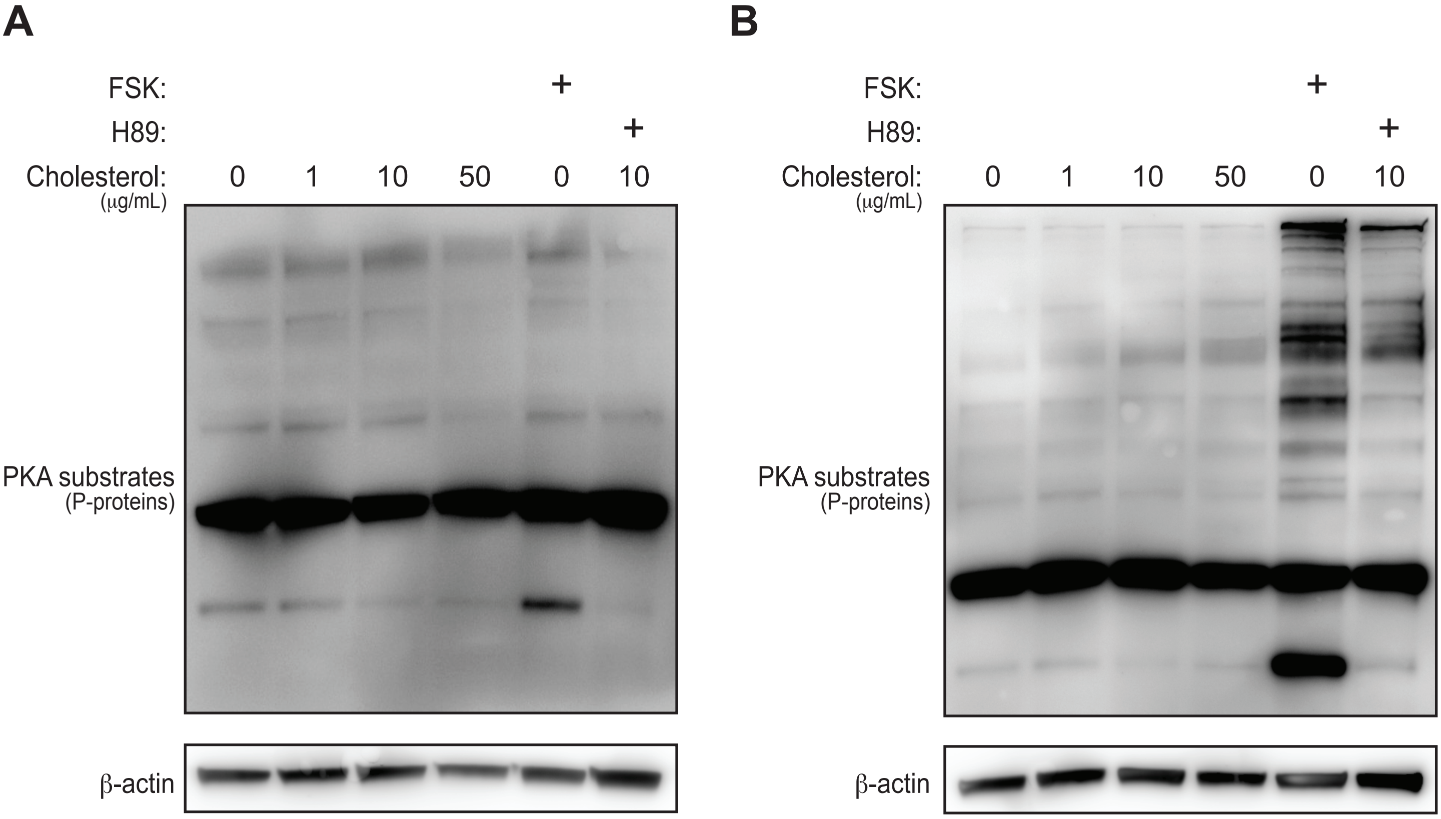

Figure S2. Cholesterol supplementation does not promotes PKA activation in pancreatic tumor cells. Pancreatic tumor cells were isolated from *Pdx1-Cre;LSL-Kras*^{G12D} (KC, panel **A**) or *Pdx1-Cre;LSL-p53*$^{fl/fl}$*;LSL-Kras*^{G12D} (KPC, panel **B**) mice. The former develop pre-neoplastic lesions (PanIN); thus KC cells are bona fide PanIN-derived cells. On the other hand, KPC mice rapidly develop PDA tumors and KPC-derived cell lines are de facto tumor cell lines. Cells were treated with free cholesterol (10 μg/mL) ± H89 or vehicle for 40 minutes. Forskolin (FSK) is an activator of PKA. Whole-cell lysates were blotted against phosphorylated PKA substrates.
